# Antibiotic-Resistant *Salmonella* in the Food Supply and the Potential Role of Antibiotic Alternatives for Control

**DOI:** 10.3390/foods7100167

**Published:** 2018-10-11

**Authors:** Divek V. T. Nair, Kumar Venkitanarayanan, Anup Kollanoor Johny

**Affiliations:** 1 Department of Animal Science, University of Minnesota, Saint Paul, MN 55108 USA; valsa002@umn.edu; 2 Department of Animal Science, University of Connecticut, Storrs, CT 06269, USA; kumar.venkitanarayanan@uconn.edu

**Keywords:** *Salmonella*, antibiotic resistant, food supply, serotypes, antibiotic alternatives, control

## Abstract

*Salmonella enterica* is one of the most ubiquitous enteropathogenic bacterial species on earth, and comprises more than 2500 serovars. Widely known for causing non-typhoidal foodborne infections (95%), and enteric (typhoid) fever in humans, *Salmonella* colonizes almost all warm- and cold-blooded animals, in addition to its extra-animal environmental strongholds. The last few decades have witnessed the emergence of highly virulent and antibiotic-resistant *Salmonella*, causing greater morbidity and mortality in humans. The emergence of several *Salmonella* serotypes resistant to multiple antibiotics in food animals underscores a significant food safety hazard. In this review, we discuss the various antibiotic-resistant *Salmonella* serotypes in food animals and the food supply, factors that contributed to their emergence, their antibiotic resistance mechanisms, the public health implications of their spread through the food supply, and the potential antibiotic alternatives for controlling them.

## 1. Introduction

Antibiotic resistance is a global phenomenon resulting in the emergence of pathogens with resistance to clinically important antibiotics, necessitating new treatment strategies [1]. Antibiotic-resistant bacteria cause life-threatening illness in humans and pose a significant threat to health and well-being. It is estimated that antibiotic-resistant pathogens cause ~2 million illnesses and 23,000 deaths annually in the U.S. These illnesses cause an additional healthcare cost of $20 billion and a productivity loss of $35 billion to the U.S. economy. Also, extensive use of antibiotics predisposes individuals to other serious illnesses, such as the *Clostridium difficile* infections that result in an estimated 250,000 infections and 14,000 deaths, annually [2]. 

Antibiotic resistance in foodborne pathogens such as *Salmonella* is a major concern for public health safety. More focus is required to target them in the animal foods supply [2]. *Salmonella* is difficult to eliminate from its reservoir hosts, and food animals often serve as reservoirs of the pathogen. Non-typhoidal *Salmonella* causes the highest number of illnesses, hospitalizations, and deaths associated with foodborne illness [3]. It is associated with more than 1,200,000 illnesses annually, and among these at least 100,000 infections are due to antibiotic-resistant *Salmonella*, including those that are resistant to clinically-important drugs such as ceftriaxone (36,000 illnesses/year) and ciprofloxacin (33,000 illnesses/year) [2]. In fact, *Salmonella* isolates conferring resistance to ≥5 antibiotics accounted for more than 66,000 illnesses from 2009 to 2011 in the U.S. [2]. 

*Salmonella* is a Gram-negative, facultatively anaerobic bacillus belonging to the *Enterobacteriaceae* family. The genus *Salmonella* is composed of two taxonomical species, *Salmonella bongori*, and *Salmonella enterica*, with all medically relevant salmonellae a part of the latter. *Salmonella enterica* is a diverse species of bacteria consisting of more than 2500 different serovars. The pathogen can be host-adapted, host-restricted, or generalistic, depending on the broad range of hosts that it can infect. The pathogen is ubiquitously present in the human food chain, and is frequently associated with outbreaks of foodborne disease. Outbreak investigations have identified food sources such as vegetables, fresh produce, cereals, cantaloupes, alfalfa sprouts, pistachios, fruit/fruit pulp, ground beef and turkeys, chicken meat and pork, tuna, dried/shredded coconut, and tomatoes as vehicles of *Salmonella*-associated foodborne outbreaks in the past decade [4]. The situation has been aggravated, since antibiotic-resistant clones are frequently implied as the etiological agents in these outbreaks leading to treatment failures, higher risk of bloodstream infections, and increased rate of hospitalizations.

Recently, drug-resistant *Salmonella* has been associated with a considerable number of outbreaks in the U.S. A non-typhoidal *Salmonella* serovar, *S.* Urbana, caused disease outbreak through papayas in 2017, and the isolates showed resistance to streptomycin and intermediate resistance to tetracycline [5]. Another serovar, *S.* Poona, caused multistate disease outbreaks through cucumbers in 2015, and the isolates were resistant to either tetracycline or nalidixic acid. In addition, the nalidixic acid-resistant isolate showed a decreased susceptibility to ciprofloxacin, a clinically important drug used in children against *Salmonella* infection [6]. Multidrug-resistant *Salmonella* I 4,[5],12:i: caused foodborne illness outbreaks through contaminated pork products in 2015, which resulted in severe infection in humans. The isolated strains were resistant to multiple antibiotics, including ampicillin, streptomycin, sulfisoxazole, and tetracycline [7]. In addition, *S.* Enteritidis isolated from raw, frozen, and stuffed chicken entrees associated with multistate disease outbreaks were resistant to ampicillin and tetracycline [8]. In 2014, *S.* Heidelberg was involved in an outbreak through mechanically separated chicken, and 67% of *Salmonella* isolates identified were resistant to three or more classes of antibiotics [9]. In addition, in 2011, multidrug-resistant *S.* Typhimurium, *S.* Heidelberg, and *S.* Hadar were associated with disease outbreaks in the U.S. through contaminated ground beef, ground turkeys, and turkey burgers, respectively [10,11,12]. These reports indicate the frequent involvement of antibiotic-resistant *Salmonella* in the human food chain, necessitating the exploration of novel non-antibiotic interventions to counteract the pathogen in reservoirs, including food animals. 

## 2. Emergence and Spread of Antibiotic-Resistant Bacteria

Antibiotics are used in food animal production to promote growth and to prevent, (prophylactic), treat (therapeutic), and control (metaphylactic) infectious diseases [13,14]. Previous studies indicated that the use of antibiotics for non-therapeutic purposes in poultry, swine, and cattle outweighed what has been used in humans by several-fold with respect to the amount of drugs consumed [13,15]. The extensive use of antibiotics in the animal production systems for the purposes mentioned above has also contributed to the development of drug-resistant bacteria. The close association of these bacteria has also been identified in the human food chain. For example, drug-resistant bacteria have been identified from various environmental samples, farms, and retail meat products [13,14,16,17,18,19]. In addition, the non-judicious use of antibiotics has been attributed to foodborne disease outbreaks where the etiological agents have been identified as resistant clones. Although a mandatory withdrawal period is necessary for avoiding the deposition of antibiotic residues in meat, milk, and eggs, lack of proper monitoring could result in residue deposition in the human food chain, resulting in the colonization of resistant bacteria in the human digestive tract [20]. 

A variety of microorganisms are present outside the host, including those found in water, soil, air, and other related environments. These environmental microorganisms are excellent sources of antimicrobial resistance genes (“environmental resistomes”). “Resistome” is a broad term that describes the presence of all antibiotic resistance genes found in free-living organisms in the environment or commensal microbes. The resistome plays a critical role in transferring antimicrobial resistance to pathogenic microorganisms, and directly affects human health by entering the food chain [21]. Studies have revealed that commensal bacterial species such as the lactic acid bacteria carry resistance genes and might serve as reservoirs of resistant genes for entero-pathogens. For example, tetracycline, vancomycin, and erythromycin resistance genes have been identified from lactic acid bacteria isolated from fermented dairy products, sausages, and raw meat products including poultry, beef, and pork [22]. 

The interaction between the different components in a food chain or the environment further contributes to the spread of antibiotic resistance across species [14]. Although humans contract infections from farm animals, pets, fresh produce, meat, eggs, and other agricultural and non-agricultural food products, there are multiple entry routes for pathogens to these vehicles [14]. Foodborne pathogens such as *Salmonella* enter a farm from different sources, such as water, litter, personnel, equipment, vehicles, rodents, insects, and pets. In addition, the movement of portable equipment and vehicles can act as a vector for carrying the pathogen to the farm environment or slaughterhouse [23]. Similarly, antibiotic-resistant bacteria also spread through truck washing systems, lairage, barn floor, barn flush, and holding pens, and potentially end up in animal carcasses during slaughter [24]. Irrespective of the antibiotic use, antibiotic-resistant pathogens such as *S.* Typhimurium have been recovered from swine and poultry housed in antibiotic-free production systems, highlighting the possible role of environmental factors and vectors such as rodents, insects, and birds in spreading resistance [14,17,18,19]. 

The fecal excretion of antibiotic-resistant pathogens such as *Salmonella* from livestock and poultry causes the contamination of the farm environment and water systems. Faulty municipal drainage systems could also result in the spread of resistant bacteria from humans to the waterways and the environment [25]. The use of fecal waste as manure in agricultural lands also contributes to the spread of antibiotic resistance, especially in fresh produce. Antibiotic-resistant foodborne pathogens such as *Salmonella*, *E. coli*, and *Shigella* have frequently been recovered from fresh produce locally grown in the U.S. or imported from other countries [26]. The application of pesticides, soil contaminated with livestock feces, and the spraying or irrigation of contaminated water cause the spread of resistant bacteria to fruits, vegetables, and fresh produce [27]. The contamination of waterways also contributes to the pool of resistant bacteria in agriculture and aquaculture. Aquaculture isolates have shown similar resistance patterns to the isolates recovered from terrestrial agriculture, indicating possible contamination of water sources from farmland [25]. 

The development and spread of antibiotic resistance are complicated processes involving different components of the human food chain, and could be a result of the intense use of antibiotics in food animal agriculture, in addition to other contributors. With such severe concerns of antibiotic resistance development in various pathogens, including the emerging multidrug-resistant strains, the Food and Drug Administration (FDA) has recently introduced the Veterinary Feed Directive (VFD) that necessitates the supervision of veterinarians before using clinically-important antibiotics in treating production animals [28,29]. The VFD highlights the importance of the judicious use of antibiotics in animal agriculture and demands the development of natural, safe, environmentally-friendly intervention strategies against deadly foodborne pathogens, including *Salmonella* [28,29]. 

## 3. Antibiotic-Resistant *Salmonella* in the Food Supply

### 3.1. Poultry and Poultry Products

Antibiotic-resistant *Salmonella* is a significant concern in poultry production. After the approval of fluoroquinolones (enrofloxacin and sarafloxacin) in poultry husbandry in 1995, an extensive use of antibiotics started to augment poultry production. However, the reports from the National Antimicrobial Resistance Monitoring System (NARMS) presented incidences of the isolation of antibiotic-resistant *Salmonella*, eventually culminating in the withdrawal of major antibiotics such as fluoroquinolones from poultry production [30,31]. Interestingly, even after the withdrawal of some of these antibiotics from production, a high prevalence of *Salmonella* resistant to fluoroquinolones has been reported, posing a significant threat to poultry food safety and human health [2,6,32,33,34].

Often, farm environments are the reservoirs of pathogens, including antibiotic-resistant bacteria [35,36]. Recently, *Salmonella* isolates resistant to multiple antibiotics, including streptomycin (30.9%), gentamicin (12.6%), sulfadimethoxine (20.9%), tetracycline (13.9%), and trimethoprim-sulfamethoxazole combination (8.6%) were recovered from broiler farms. Among these isolates, 20% were resistant to three or more antibiotics; 67% of *S.* Heidelberg and 54% of *S.* Kentucky isolates showed resistance to five or more antibiotics [37]. In addition to a high prevalence of *S.* Enteritidis noticed in hatching eggs, litter, feed, drinkers, bird rinse, and ceca, 88% of *S.* Enteritidis were reported to be resistant to multiple drugs including ampicillin, nalidixic acid, and tetracycline [32]. 

Currently, intervention strategies are practiced at the farm level to control antibiotic-resistant *Salmonella* in poultry and its spread to carcasses during processing. However, antibiotic-resistant strains of *Salmonella* serovars such as *S.* Enteritidis, *S.* Infantis, *S.* Typhimurium, and *S*. Heidelberg have frequently been isolated from broiler carcasses [38]. Augusto et al. [38] reported high resistance of the aforementioned isolates towards ceftriaxone (75%) and ceftiofur (44%). Recently, a Canadian study reported a significant correlation between the isolation of ceftiofur-resistant *S.* Heidelberg from retail chicken meat and the incidence of human clinical infections with *S.* Heidelberg during 2003–2008 [39]. Another study conducted by Duiy et al. [40] revealed a high prevalence of antibiotic-resistant *Salmonella* in poultry meat compared to beef and lamb samples, and observed *S*. Bredeney, *S.* Kentucky, and *S*. Enteritidis as major serotypes isolated from poultry meat. These serotypes showed high resistance to antibiotics such as rifampicin, tetracycline, oxytetracycline, and sulphamethoxazole. Also, ceftiofur and ceftriaxone-resistant *Salmonella* were isolated from ground chicken and turkeys [41]. 

Antibiotic-resistant *Salmonella* has been isolated from retail meats as well. For example, sampling from poultry retail outlets of Pennsylvania during 2006–2007 showed contamination of 22% of meat samples with *Salmonella.* Among the different *Salmonella* isolates, 31% were resistant to three antibiotics, and 21% were resistant to ceftiofur. In addition, one ceftiofur-resistant *S.* Typhimurium showed an indistinguishable pulse field gel electrophoresis (PFGE) pattern with a human isolate [42]. Along with this, sampling of whole chicken carcass from the retail outlets [33] revealed a high prevalence of *Salmonella* (25%). Among these, 40% of isolates were resistant to multiple antibiotics (≥4 antibiotics), 70% were resistant to at least one antibiotic, and 52% isolates showed increased resistance to ciprofloxacin. Another study conducted in retail meats revealed *Salmonella* isolates with high resistance to common antibiotics. Among the different isolates, 82% showed resistance to at least one antibiotic, and the increased resistance observed was against tetracycline (68%), streptomycin (61%), sulfamethoxazole (42%), and ampicillin (29%) [43]. Also, 9% of the *Salmonella* isolates showed resistance to ceftriaxone. Another study conducted by Parveen and co-workers [44] revealed the presence of antibiotic-resistant *Salmonella* isolates from poultry chiller water and carcasses. The isolated *Salmonella* spp. were resistant to antibiotics including tetracycline, ampicillin, amoxicillin-clavulanic acid, ceftiofur, streptomycin, and sulfisoxazole [44]. Recently, the FDA published the NARMS retail meat interim report for *Salmonella*, which includes the antibiotic resistance profile of *Salmonella* spp. in retail poultry meat [34]. 

The intense use of antibiotics in conventional farms has promoted resistance development in *Salmonella* spp. [45]. Sapkota et al. [45] reported that when a conventional farm was converted to an organic farm, the prevalence of the antibiotic-resistant *Salmonella* was reduced. The *Salmonella* isolates, including *S.* Kentucky from the organic production facility, showed significantly lower resistance to antibiotics such as amoxicillin-clavulanate, ampicillin, cefoxitin, ceftiofur, and ceftriaxone. Among the *S.* Kentucky isolates, only 6% from the organic farm showed multiple antibiotic resistance whereas 44% of isolates from conventional farms showed multiple antibiotic resistance [45]. A study conducted by Alali et al. [17] also reported a high prevalence rate of *Salmonella* in fecal and feed samples in conventional farms compared to the certified organic facilities when the chickens were 3 and 8 weeks old. The population of resistant bacteria was higher from the conventional farm, where the resistance to a single antibiotic and two or more antibiotics was 36.2% and 62%, respectively. The isolates showed high resistance to ampicillin, streptomycin, amoxicillin-clavulanic acid, cephalothin, ceftiofur, and cefoxitin. When a comparison of *Salmonella* isolates obtained from organic and conventional poultry samples from Maryland retail stores was made, all *S.* Typhimurium isolates (12 isolates; 100%) obtained from conventional carcass samples were resistant to between five and seven antimicrobials, whereas 79% of the *S*. Typhimurium isolates (15 out of 19 total isolates) from organic carcass samples showed susceptibility to all 17 tested antimicrobials [46]. 

### 3.2. Cattle and Beef

Antibiotic-resistant *Salmonella* has also been isolated from beef cattle. A study conducted in the USA reported a high prevalence of *Salmonella* from hide and feces swab samples (70% and 30%, respectively) from cattle nearing their market age. Among the *Salmonella enterica* isolates, 33.1%, 8.35%, 3.75%, and 3.75% of isolates were resistant to one, two, three, and four or more antibiotics, respectively. Resistance to sulfisoxazole (39.5% of isolates), tetracycline (10.9%), and ampicillin (8.89%) was common. However, ceftriaxone resistance was also detected in two isolates [47]. A recent study by Schmidt et al. [48] evaluated the presence of antibiotic-resistant *Salmonella* from production to processing continuum by sampling feces, hides, carcass, and strip loins. Third-generation cephalosporin-resistant *Salmonella* was detected in 0.5% of fecal samples and 10.9% of hide samples from feedlots. In addition, prevalence rates of 1.6% and 7.6% cephalosporin-resistant *Salmonella* were observed during processing from fecal and hide samples, respectively. However, none of the pre-eviscerated carcasses, final carcasses, or strip loin samples were found to be positive for antibiotic-resistant *Salmonella*, which indicates the effectiveness of sanitation procedures during processing in preventing the hide-to-carcass transfer of antibiotic-resistant *Salmonella*. However, *Salmonella* can harbor in bovine peripheral lymph nodes such as subiliac lymph nodes, which are often protected from the carcass sanitation procedures and pose potential threats when adipose trim containing lymph nodes are incorporated into the ground beef [49]. A cross-sectional study conducted by collecting subiliac lymph nodes (*n* = 3327) from feedlot cattle at harvest revealed a 11.8% prevalence rate of *Salmonella enterica* isolates, with *S.* Montevideo and *S.* Anatum as major serovars. Among these, 8.3% were resistant to multiple antibiotics including ampicillin, chloramphenicol, streptomycin, sulfisoxazole, tetracycline, amoxicillin/clavulanic acid, ceftiofur, and ceftriaxone [49]. 

Ground beef also carries antibiotic-resistant *Salmonella.* A study conducted by White et al. [41] revealed a *Salmonella* prevalence rate of approximately 6% in ground beef samples, and the isolated *S.* Typhimurium strains were highly resistant to ceftiofur and ceftriaxone. In another study, *Salmonella* serovars such as *S.* Typhimurium, *S.* Lille, *S.* Montevideo, *S.* Hadar, *S.* Meleagridis, *S.* Cerro, *S.* Kentucky, or *S.* Muenster were identified from ground beef samples collected from 404 retail stores. Among these, five *S.* Typhimurium isolates were resistant to ampicillin, streptomycin, sulfamethoxazole, ticarcillin, and tetracycline [50]. Recently, the FDA published the NARMS retail meat interim report for *Salmonella* that includes the antibiotic resistance profile of *Salmonella* spp. in retail beef products [34]. According to the report, 38.5% of the *Salmonella* spp. isolated from ground beef in 2014 showed resistance to ceftriaxone and were resistant to three or more classes of antibiotics. In addition, the genes conferring resistance to major classes of antibiotics such as *aadB*, *strA/strB*, *blaCMY*, *sul2*, *blaTEM*, *floR*, *cmlA*, *gyrA mutation*, *tetA*, *tetB, or tetC* were detected in *Salmonella* isolates from ground beef. The role of these genes in conferring antimicrobial resistance in *Salmonella* is described later in this review [34]. 

The presence of multidrug-resistant *Salmonella* in dairy cattle is also a significant threat to food safety. A study conducted by Cobbold et al. [51] revealed a prevalence rate of 32% *Salmonella* in dairy farms, with a high prevalence for multidrug-resistant *S.* Newport. In addition, the persistent excretion of *Salmonella* in cows (for 190 days) often resulted in the contamination of farm environments such as bedding materials, feed refusals, lagoon slurry, and milk filters. In a different study, Rodriguez-Rivera et al. [52] reported the prevalence of *Salmonella* serovars such as *S.* Cerro, *S.* Kentucky, *S.* Typhimurium, and *S.* Anatum along with *S.* Newport in dairy cattle and farm environments. The same study reported that 23.6% of isolates were resistant to clinically-important antibiotics with 50 different resistance patterns, including 12 serovars showing indistinguishable PFGE patterns with human isolates, indicating the reservoir status of subclinically infected dairy cattle as a source of human salmonellosis. The most common resistance observed was to antibiotics such as ampicillin (72% of the isolates), tetracycline (63% of the isolates), and amoxicillin/clavulanic acid (58% of the isolates). Among the different *Salmonella* serovars, *S.* Typhimurium showed the highest resistance to the tested antibiotics, and *S.* Typhimurium isolates alone showed 20 different resistance patterns. The antibiotic resistance observed with the different serovars was in the following order: *S.* Typhimurium > *S.* Cerro > *S.* Newport > *S.* Kentucky. 

Studies have also revealed that the dairy herds (93 herds) in the northeastern U.S. have a *Salmonella* prevalence rate of 22.5%, with a high incidence rate for *S.* Newport (41%) and *S.* Typhimurium. The isolates showed higher resistance to clinically important drugs such as ampicillin (68.8%), ceftiofur (60.4%), chlortetracycline (66.8%), florfenicol (63.7%), neomycin (42.2%), oxytetracycline (68.9%), and sulfadimethoxine (79.3%). Among the different isolates, *S.* Newport (97% isolates), *S.* Typhimurium (Copenhagen) (98.4% of isolates), and *S.* Agona (83.3% of isolates) showed resistance to five or more antibiotics [53]. *S.* Typhimurium isolates from cattle were found to be associated with *Salmonella* infection in humans, and were resistant to ampicillin, sulfisoxazole, kanamycin, and streptomycin, as well as to broad-spectrum cephalosporins, aztreonam, cefoxitin, gentamicin, and tobramycin [54].

### 3.3. Swine

Similar to poultry, antibiotic usage started in swine production as early as the 1950s. Antibiotics including tetracycline, sulfonamides, and bacitracin were commonly used in swine production to increase the production performance or as therapeutic agents. Also, antibiotics were found to be an effective remedy against mortality and morbidity in young pigs and piglets to diseases [55]. Ever since the introduction of antibiotics, the extensive use of antibiotics has resulted in the development of resistance in human pathogens, including *Salmonella* in swine [18,19,56]. Both swine-adapted and non-swine-specific serovars of *Salmonella* developed antibiotic resistance [57]. *S. cholerasuis* is a swine-adapted serovar of *Salmonella* that causes severe invasive infections in humans, and often requires antibiotic treatment. This particular serovar developed antibiotic resistance against clinically important drugs as evidenced by a study conducted by Lynne et al. [58]. The results of the study revealed that 87.5% of swine isolates of *S. cholerasuis* were resistant to at least one antibiotic, whereas 37.5% showed resistance to four or more antibiotics. The most common resistance pattern observed was against tetracycline, ampicillin, streptomycin, and sulfisoxazole [58]. The resistance development in *S. cholerasuis* is a serious concern, since 52% of culture-confirmed cases of *S. cholerasuis* are linked with human infections [59]. Furthermore, the population dynamics of antibiotic-resistant *Salmonella* serovars vary in swine due to the varying selection pressure exerted by the different antibiotics [57]. Therefore, a constant monitoring system is required to detect the prevalence of antibiotic-resistant *Salmonella* in swine production. 

Multidrug-resistant *Salmonella* has been identified from intensive and extensive rearing systems in swine herds. High resistance profiles against tetracycline (78.5%) and streptomycin (31.5%) have been reported when environmental and carcass swabs from extensive and intensive poultry farming facilities were sampled. Among the different serovars, *S.* Typhimurium var. Copenhagen showed resistance against ampicillin, chloramphenicol, streptomycin, sulfamethoxazole, and tetracycline [19]. Similarly, Gebreyes et al. [18] also reported antibiotic-resistant *Salmonella* isolates in conventional farms compared to antibiotic-free non-conventional farms with high resistance to tetracycline (80%). 

Baggesen and Aarestrup [60] reported multiple-antibiotic-resistant *Salmonella* from swine herds. Among the 670 isolates, 34% were resistant to streptomycin, and 17% were resistant to tetracycline. In addition, multidrug-resistant *S.* Typhimurium DT104 was isolated from the swine herds conferring resistance to spectinomycin, streptomycin, and sulphonamides. Similarly, Perron et al. [61] reported that *Salmonella* serovars from pigs were resistant to common antibiotics and 65% of isolates showed resistance to tetracycline, whereas 25% of all isolates were multidrug resistant. Moreover, 90% of *S.* Typhimurium DT104 were resistant to 2 or more antibiotics. In this study, the detected antibiotic resistance was in the following order: tetracycline > ampicillin > chloramphenicol > neomycin > trimethoprim-sulfa combination. 

Similar to poultry and cattle, antibiotic-resistant *Salmonella* were isolated from feces, cecal contents, and mesenteric lymph nodes of pigs, and in environmental samples such as barn floor, lagoon, barn flush, trucks, and holding pens [24]. Multidrug resistance patterns such as AxACSSuT (amoxicillin-clavulanic acid, ampicillin, chloramphenicol, streptomycin, sulfamethoxazole, and tetracycline) and AKSSuT (ampicillin, kanamycin, streptomycin, sulfamethoxazole, and tetracycline) were common in 36% and 45% of the *S.* Typhimurium isolates from swine feces, respectively [24]. Also, high resistance to tetracycline (85%), ampicillin (47%), amoxicillin-clavulanic acid (23%), and chloramphenicol (21%) was reported in the same study [62]. Another serovar of *Salmonella*, *S.* Muenchen, was also identified from pigs, and 75% of the isolates showed AKSSuT-type resistance [63]. Also, improperly decontaminated swine manure application was found to be another important source of dissemination and persistence of antibiotic-resistant *Salmonella* in the environment. High frequency of resistance against streptomycin (88.36%), sulfisoxazole (67.2%), and tetracycline (57.67%) was identified in swine manure samples [56].

Antibiotic-resistant *Salmonella* has frequently been isolated from retail pork products. Pork chops and pork ribs carrying antibiotic-resistant *Salmonella* containing *blaCMY* (gene encoding β-lactam resistance) has been identified [64]. Recently, the FDA published the NARMS retail meat interim report for *Salmonella* that includes the antibiotic-resistance profile of *Salmonella* spp. in retail pork products [34]. 

### 3.4. Fresh Produce 

Antibiotic-resistant pathogens have also been isolated from fresh produce, although it is less frequent as compared with that recovered from food animals. The U.S. domestic market has a high demand for fresh produce. Billions of dollars worth of fresh produce, fruits, and vegetables are exported from ($7 billion) and imported to ($18 billion) the U.S. [65]. Fresh produce such as leafy greens, herbs, and spinach in retail stores are often contaminated with coliform bacteria. Aerobic plate counts as high as 6–7.4 log_10_ CFU/g and a coliform count ranging from 0 to >8.5 log_10_ CFU/g have been reported previously [66]. On the other hand, recent studies have revealed a low prevalence of *Salmonella* in fresh produce [26,66]. Liu and Kilonzo-Nthenge [26] reported that the prevalence of *Salmonella* in fresh produce from the U.S. chain markets (a total of 360 fresh produce samples; 129 imported and 231 U.S.-grown) was 0.3%, whereas 0.8% fresh produce imported to the U.S. was positive for *Salmonella.* The antibiotic resistance was reported in *Salmonella* isolates recovered from imported fresh produce (1.9% of the isolates). The majority of them showed resistance to ampicillin-erythromycin-kanamycin (AEK) and streptomycin-vancomycin (SV) resistance patterns [26]. 

Environmental contamination may have a direct relationship to the presence of antibiotic-resistant *Salmonella* in fresh produce. For example, studies by Duffy et al. [67] indicated *Salmonella* prevalence in irrigation water, packing shed equipment, and fresh produce from two different produce farms. Among these, 20% of the *Salmonella* isolates showed intermediate resistance to streptomycin. Similarly, *Salmonella* isolates from cantaloupes showed resistance to streptomycin. Irrigation water is also a source of the contamination of fresh produce with *Salmonella*. *Salmonella* serovars such as *S*. Newport, *S.* Enteritidis, *S.* Muenchen, *S.* Javiana, and *S.* Thompson have been isolated from ponds in fresh produce farms. *S.* Newport was resistant to multiple antibiotics, and some of them showed PFGE patterns identical to human isolates obtained from clinical settings. *S.* Newport isolates showed resistance to ampicillin, chloramphenicol, streptomycin, sulfamethoxazole, and tetracycline (ACSSuT) and also to cephalothin, ceftriaxone, and amoxicillin-clavulanic acid [68].

The intrusion of wild animals into crop fields often introduces pathogens, especially antibiotic-resistant clones, posing a threat to food safety. A prevalence study conducted on the feces of stray dogs and coyotes (461 fecal samples) collected from the leafy green fields at the U.S.–Mexico border revealed a *Salmonella* prevalence rate of 9% and 32% in dog and coyote fecal samples, respectively. Among *Salmonella* isolates, 12% were resistant to at least one antibiotic, and 6% were resistant to two or more antibiotics. *S.* Newport isolated from coyote fecal samples showed antibiotic resistance to multiple drugs, such as ampicillin, amoxicillin/clavulanic acid, ceftriaxone, chloramphenicol, and trimethoprim/sulfamethoxazole [69]. Wild amphibians and reptiles associated with surface waters in crop-producing regions can also act as a source of pathogens on fresh produce. Among the 460 amphibians and reptiles sampled, 37 were found to harbor *Salmonella*, with snakes having a high prevalence rate of 59%. In addition, 12.6% of the water samples were positive for *Salmonella.* In the same study, PFGE revealed the occurrence of similar *Salmonella* strains in water and animal samples. Among the 66 total *Salmonella* isolates, 23 were resistant to more than one class of antibiotics, whereas six isolates were resistant to three classes of antibiotics. *S. enterica* subspecies IIIb 38:l, v:z_53_ from snake was the most resistant isolate, and showed resistance to amikacin, gentamicin, streptomycin, cephalothin, and ampicillin. In addition, the isolated *S. enterica* subspecies IIIa and IIIb were previously associated with human illnesses [70]. The domestic animals are also involved in the environmental contamination with antibiotic-resistant *Salmonella*, and subsequently contaminate vegetables. For example, dairy and beef cattle can excrete *Salmonella* through their feces, which can contaminate the crops when used as manure without appropriate treatments [47,48,71]. Therefore, the role of domestic species and wild animals in serving as reservoirs of antibiotic resistance clones to crops and vegetables needs to be explored to provide the scientific basis for effective preventative measures. 

Recently, drug-resistant *S.* Poona caused a multistate outbreak in the U.S. involving 40 states due to the consumption of contaminated garden cucumber, resulting in 204 hospitalizations and six deaths during 2015–2016. Among the isolates that were identified as etiological agents, two were resistant to antibiotics. One isolate was resistant to tetracycline, and the other was resistant to nalidixic acid and ciprofloxacin [6]. The outbreak investigation could not determine an association of the illness with cross-contamination within the distribution chain, including shipping containers or retail outlets. Therefore, the presence of antibiotic-resistant *Salmonella* in fresh produce and its association with foodborne outbreaks warrants stricter surveillance and targeted interventions. 

### 3.5. Seafood

Seafood is also a commonly implicated vehicle for the transmission of antibiotic-resistant bacteria [72,73,74], and imported seafood in the U.S. contributes to more than 80% of the supply. Foodborne pathogens such as *Salmonella*, *Listeria monocytogenes*, *Campylobacter*, and *E. coli* have been isolated from imported seafood [75]. A study conducted in the U.S. revealed the prevalence of antibiotic-resistant *Salmonella* (24%) in imported seafood. The same study identified *S.* Newport, *S.* Typhimurium var. Copenhagen and *S*. Lansing with resistance to multiple antibiotics, including trimethoprim-sulfamethoxazole combination, sulfisoxazole, tetracycline, streptomycin, and spectinomycin. Among the different isolates, 6% were resistant to more than two antibiotics [73]. Another study reported that the presence of antibiotic resistance was higher in farm-raised shrimps as compared to wild-caught shrimps. In farm-raised shrimps, 9.8%, 3.6%, and 10.5% of *Salmonella* isolates showed reduced susceptibility to the antibiotics such as ceftriaxone, chloramphenicol, and tetracycline, respectively whereas, in wild-caught shrimps, 2.8, 1.4 and 1.1% bacterial isolates showed reduced susceptibility to these antibiotics. One *Salmonella* isolate from farm-raised shrimps was resistant to multiple antibiotics, including ampicillin, ceftriaxone, gentamicin, streptomycin, and trimethoprim [76].

Similarly, a study conducted in oysters harvested from U.S. bays revealed the prevalence of *Salmonella* (7.4%), with *S.* Newport as the major serovar. Most of the *Salmonella* isolates from oysters were resistant to ampicillin and tetracycline [74]. In addition to raw sources, ready-to-eat (RTE) shrimp is one of the vehicles for spreading antibiotic-resistant *Salmonella.* A study by Dur et al. [77] revealed that RTE shrimps contained *Salmonella* as an adulterant and the isolated strains were less susceptible to commonly used antibiotics. The RTE shrimps could be contaminated with *Salmonella* during post-processing steps such as thawing [77]. Furthermore, a recent multistate outbreak of *S.* Paratyphi B and *S.* Weltevreden was reported in the U.S. in 2015 involving 11 states, affecting 65 people, and causing 11 hospitalizations. The infection was linked to contaminated frozen raw tuna. Among the *Salmonella* isolates, 33% showed resistance to ampicillin. However, others were pan-susceptible [78]. 

## 4. Mechanisms of Antibiotic Resistance in *Salmonella* and Public Health Implications

The horizontal transmission of resistance genes plays a vital role in the dissemination of antibiotic resistance in *Salmonella enterica* species. These resistance genes can be found in the resistant plasmids or within the chromosome of bacteria. The horizontal transmission of genes mediated by plasmids is the most efficient method of resistance transfer, and is occurring at high frequency involving different resistance genes at a time [79]. The resistant genes that are acquired by plasmids, integrons, or transposons are capable of transferring resistance to other strains or other species. Transposons are the mobile genetic elements that can carry resistance genes and possess transposase activity providing the recombination of resistance genes with plasmids or the chromosome. Integrons consist of integrase (a recombination enzyme encoded by the *intI* gene), a recombination site recognized by integrase, and a promoter which are necessary for the expression of gene cassettes present in the integron [80]. These arrangements efficiently promote the acquisition of exogenous genes such as antibiotic-resistant genes in the bacterial genome, especially in plasmids. Furthermore, the conjugation events facilitate the spread of resistance genes present in plasmids through transposon or integron to other strains or species [79]. 

The emergence of *S.* Typhimurium definitive type (DT)104 as a multidrug-resistant pathogen was a significant issue in animal agriculture. It was first isolated from the United Kingdom, and since then it has been associated with monogastric animals and ruminants, causing foodborne outbreaks through meat and meat products. The chromosomally encoded resistance to ≥5 antibiotics, including ampicillin, chloramphenicol, florfenicol, streptomycin, sulfonamides, and tetracyclines, makes this phage type challenging to tackle. The trimethoprim resistance in *S.* Typhimurium DT104 has been found to be associated with mobile non-conjugative plasmids [81]. 

The resistance of non-typhoidal *Salmonella* to fluoroquinolones is of particular concern since it is the drug of choice to treat invasive salmonellosis in adults. The fluoroquinolone resistance was previously linked to multiple mutations (e.g., amino acid substitutions) in quinolone resistance-determining regions (QRDRs) of the genes that code for gyrase (*gyrA* and *gyrB*) and topoisomerase IV, which are the targets for fluoroquinolones in bacterial cells. The mutation of these genes results in resistance to fluoroquinolones [81,82]. Also, the presence of an active efflux pump was reported in *S.* Typhimurium as a mechanism of antibiotic resistance. The overproduction of AcrAB (inner membrane transporter)-TolC (outer membrane transporter)-type efflux pump and associated alterations in outer membrane proteins and lipopolysaccharides synergistically caused less accumulation of ciprofloxacin in *S.* Typhimurium, and showed increased resistance in *Salmonella.* However, efflux pump blockers significantly increase the susceptibility (16–32 times) of *Salmonella* to fluoroquinolones [81,83]. 

Plasmid-mediated quinolone resistance (PMQR) genes are also involved in resistance build-up in *Salmonella.* PMQR genes such as *oqxAB* and *aac(6’)-Ib-cr* are isolated with high frequency (44% and 89%; *oqxAB* and *aac(6’)Ib-cr*, respectively) from ciprofloxacin-resistant clinical isolates of *S.* Typhimurium. These PMQR genes along with *gyrA* mutations increase the minimum inhibitory concentration of ciprofloxacin by four-fold in *S.* Typhimurium. Among other PMQRs, *qnr* type genes also bind to DNA gyrase and topoisomerase and prevent the action of fluoroquinolones. Another PMQR gene, *qepA*, is associated with efflux pump and excretes fluoroquinolones to the extracellular space. However, further studies are needed to establish their prevalence in non-typhoidal *Salmonella* [84]. As mentioned, the AcrAB-TolC efflux system and its regulatory genes such as *marRAB* and *soxRS* are found to be involved in fluoroquinolone resistance which increased the minimum inhibitory concentration (MIC) of fluoroquinolones to ≥32 µg/mL in *S.* Typhimurium phage type DT204 [85] and the inactivation of the efflux pump resulted in a 16–32-fold reduction of the MIC of *S.* Typhimurium phage type DT204 to ciprofloxacin [86]. Therefore, the resistance of non-typhoidal *Salmonella* to fluoroquinolones is often attributed to a combination of mechanisms [87]. 

Non-typhoidal *Salmonella* spp. showing resistance to extended-spectrum cephalosporins, including ceftriaxone, is a serious concern, since these are the drugs of choice for treating invasive non-typhoidal salmonellosis in children. One of the major mechanisms of developing resistance against β-lactam antibiotics in bacteria is the direct inactivation of antibiotics by enzyme hydrolysis [88]. The production of extended spectrum β-lactamases (ESBLs) is a major mechanism conferring resistance in most of the *Enterobacteriaceae.* Many types of ESBLs are present based on the substrate and inhibitor mechanisms [89]. The first β-lactamase identified was TEM-1 found in an *E. coli* strain isolated from a patient named Temoniera in Greece [90]. TEM-1 hydrolyzes penicillins and first-generation cephalosporins. A single amino acid substitution to TEM-1 leads to a TEM-2 derivative having the same substrate as that of the TEM-1. The first TEM-type β-lactamase that demonstrated ESBL characteristics was TEM-3 [89]. The TEM-type β-lactamases are reported in *Salmonella* spp. [91,92]. Another β-lactamase, SHV (sulphydryl variable) is a plasmid-encoded β-lactamase usually found in *Klebsiella pneumoniae* and *E. coli* [90]. The TEM and SHV types of β-lactamases are most common, and are widely distributed in nature with more than 90 types of TEM and more than 25 types of SHV [90,92].

Recently, the emergence of plasmid-mediated ESBLs, namely CTX-M, is of significant concern since it is commonly found in *Salmonella* spp. and associated with cefotaxime hydrolysis. The horizontal transfer of CTX-M ESBL genes via conjugation plasmids and transposons are the main process involved in the acquisition of CTX-M ESBLs. The expansion of CTX-M-type β-lactamase has not been explored much, and has been different from TEM- and SHV-type β-lactamases where the amino acid substitutions are common [89]. However, it has been suggested that serine residue present at position 237 in all CTX-M type enzymes plays a role in displaying extended-spectrum antibiotic resistance [93]. In *Salmonella*, most of the ESBLs (e.g., *blaTEM*-1 and *blaSHV*-1 gene derivatives), including enzymes conferring resistance to third-generation cephalosporins such as *blaCTX*-M and *blaSHV*-5, are encoded on transferable plasmids that pose a serious threat to current antibiotic treatment strategies in humans [79].

Another class of β-lactamases is the OXA type that confers resistance to ampicillin and cephalolecithin and also possesses strong hydrolytic activity against oxacillin and cloxacillin [94]. OXA-48 carbapenemase-producing *Salmonella* (*S*. Kentucky) and OXA-1 encoding poultry isolates have been identified [91,95]. PER-type ESBLs (first discovered in *Pseudomonas aeruginosa* strains) hydrolyzing penicillins and cephalosporins have also been reported in non-typhoidal *Salmonella* [96]. In addition, intrinsic cephalosporinases such as AmpC-type β-lactamases are also common in non-typhoidal *Salmonella* which includes enzyme types such as CMY, DHA, and ACC-1 [81,96]. Moreover, *Salmonella* serovars exhibiting different β-lactamases such as CMY-7, SHV-9, and OXA-30 were also identified [97], indicating the possession of a high level of cross-resistance by non-typhoidal *Salmonella* serovars.

Aminoglycoside-modifying enzymes mainly mediate resistance to aminoglycoside antibiotics. The aminoglycoside acetyltransferases modify amino groups in aminoglycoside antibiotics. The genes encoding aminoglycoside acetyltransferases are named as *aac*, and are typically located in *Salmonella* genomic islands, integrons, and plasmids. These acetyltransferases provide resistance to major antibiotics such as gentamicin and kanamycin. In addition, aminoglycoside hydroxyl group phosphorylating enzymes, namely aminoglycoside phosphotransferases, are involved in resistance development against aminoglycoside antibiotics in *Salmonella.* These enzymes are encoded by the genes *strA*, *strB*, *aph(3)-Ib* and *aph(6)-Id*, respectively) and provide resistance to streptomycin. Some of the aminoglycoside phosphotransferases also provide resistance to kanamycin and neomycin. Nucleotidyltransferases are also hydroxyl group-modifying enzymes present in *Salmonella* and are often encoded in *aad* genes. Among the different varieties of aminoglycoside adenylyltransferase coding genes, *aadA* provides resistance to streptomycin whereas *aadB* provides resistance to gentamicin and tobramycin in *Salmonella* [98]. 

Tetracycline resistance is mainly developed in *Salmonella* due to the acquisition of genes that code for energy-dependent efflux mechanisms [98]. Mainly *tet genes* are involved in efflux mechanisms, and confer resistance to chlortetracycline, doxycycline, oxytetracycline, and tetracycline [99]. Among these, *tet(A)* is common. However, others such as *tet(B)*, *tet(C)*, *tet(D)*, *tet(G)*, and *tet(H)* have been reported in non-typhoidal *Salmonella* from clinical or retail meat isolates [98,100]. The *tet(A)* genes have been found in plasmids, integrons, and genomic island 1. The *tet(B)* are detected in transferable plasmids. The *tet(A)* genes are detected in *Salmonella* serovars such as *S.* Agona, *S.* Dublin, *S.* Choleraesuis, *S.* Heidelberg, and *S.* Typhimurium [98]. In addition to this, ribosomal protection proteins such as *tet(M)*, *tet(O)*, *tet(S)*, *tet(W)*, and *tet(32)* prevent the ribosomes from the action of tetracyclines in microorganisms. Some genes encode enzymes such as *tet(X)*, *tet(34)*, and *tet (37)* which modify or inactivate the action of tetracyclines. However, the efflux mechanisms are more common [99]. 

The resistance development in microorganisms against phenicol antibiotics including chloramphenicol and florfenicol is mainly by two mechanisms involving efflux pumps or enzymatic inactivation of antibiotics by chloramphenicol O-acetyltransferase. The chloramphenicol O-acetyltransferase enzymes are not capable of inactivating florfenicol since the fluorinated c3 position in florfenicol does not accept acetyl groups. The genes encoding chloramphenicol O-acetyltransferases are referred to as *cat* genes and are often associated with plasmids. The *cat1* and *cat2* genes have been isolated from non-typhoidal *Salmonella* serovars. The *cat* genes are associated with plasmids, transposons, or gene cassettes and other mobile genetic elements [101]. Genes such as *cmlA* and *floR* encode the efflux pumps in *Salmonella* isolates. The *floR* genes are widely distributed among the *Salmonella* serovars and are found to be associated with transferable plasmids and *Salmonella* genomic islands [98]. 

The sulfonamide resistance in *Salmonella* is due to the presence of the *sul* gene, which causes the expression of an insensitive form of dihydropteroate synthetase that cannot be inhibited by sulfonamides. The common *sul* genes are *sul1*, *sul2*, and *sul3*, which have been identified from major *Salmonella* serovars, including *S.* Enteritidis, *S*, Typhimurium, *S.* Heidelberg, and *S.* Hadar. These genes are present in integrons, *Salmonella* genomic islands, or transferrable plasmids [98,102]. The *dhfr* genes cause the expression of an insensitive form of dihydrofolate reductase (DHFR) that cannot be inhibited by trimethoprim antibiotics and lead to the development of resistance against this class of antibiotics in *Salmonella.* These genes are also associated with integrons, plasmids, or *Salmonella* genomic islands [98,102]. 

## 5. Antibiotic Alternatives against *Salmonella*

Not many interventions have targeted antibiotic-resistant *Salmonella*. Although it is generally understood that the interventions used against the non-antibiotic-resistant bacteria could work equally well against resistant *Salmonella*, this area needs further exploration. Given below are the potential interventions that could be used to target antibiotic-resistant *Salmonella*. The interventions explained here are mostly targeted to control antibiotic-resistant *Salmonella* colonization in food animals and poultry for improving the preharvest microbiological safety with some directions for potential postharvest applications. 

### 5.1. Direct Fed Microbials (DFMs)

Interventions using DFMs and probiotic bacteria are getting widespread support due to the current issues associated with antibiotic resistance and the emergence of organic production as a significant contributor to the domestic and international markets [103,104]. Probiotics, including DFMs, are used/tested to maintain balanced microbial ecology in the gut [105], enhance the production performance of livestock, and as potential antimicrobials against pathogenic bacteria [106]. Probiotics such as *B. subtilis*, *Lactobacillus* strains, *Saccharomyces* (probiotic yeast), and *Aspergillus oryzae* have antimicrobial properties against pathogenic bacteria including *Salmonella* spp. [104,107,108,109], and among these, *Lactobacillus* strains are being generally used in animal agriculture [110]. 

Different strains of *Lactobacillus* species are commonly used in poultry to reduce cecal colonization of *Salmonella* and its fecal shedding. Spray application and drinking water supplementation of a *Lactobacillus*-based probiotics were also found to be effective in reducing *S.* Enteritidis colonization in cecal tonsils in broiler chicks [111]. A study conducted by Higgins et al. [112] revealed that the oral administration of *Lactobacillus* spp. at concentrations of 10^6^ and 10^8^/chick resulted in significant reduction of *S.* Enteritidis after experimentally challenging the neonatal broiler chicks with 10^4^ cfu *S.* Enteritidis orally. The recovery rate of *Salmonella* in the treatments was 15%, whereas a higher recovery of 85% was obtained in the control group. Another study conducted by Menconi et al. [113] described the efficacy of a *Lactobacillus*-based probiotic, namely FloraMax, in reducing the cecal colonization of *S.* Heidelberg after 24 and 72 h post-infection in experimentally challenged chicks and poults. Also, studies showed that *Lactobacillus salivarius* CTC2197 could eliminate *S.* Enteritidis from chickens after an oral challenge of the pathogen on day 1 [114]. Some strains of *Bacillus subtilis* were found effective against *S.* Enteritidis attachment and invasion of intestinal epithelial cells [115]. 

In swine, DFM supplementation was found to be effective against *Salmonella.* A combination of *Bifidobacterium longum* subsp. *infantis* and *B. animalis* subsp. *lactis* resulted in reduced colonization of *S.* Typhimurium in weaned piglets. The DFM combination resulted in improved intestinal health and reduced the fecal shedding of *Salmonella* after 4 and 8 days post-challenge [116]. Prophylactic administration of *L. rhamnosus* GG reduced *S.* Infantis-induced diarrhea and intestinal inflammation in piglets [117]. A study conducted by Yin et al. [118] revealed that *Lactobacillus*-fermented feed significantly reduced intestinal colonization of *S.* Typhimurium DT104 and associated diarrhea in pigs and reduced the invasion of *Salmonella* to the spleen. Also, some marine isolates of *Bacillus* strains possessed excellent probiotic qualities and *Salmonella* inhibition activities. These strains could be potential probiotics for livestock [119]. Similar to poultry and swine, DFMs such as *P. freudenreichii*, *L. animalis*, *L. acidophilus*, *L. casei*, *L. salivarius*, and, *Pediococcus acidilactici* were found to be effective against major serovars of *Salmonella* colonizing in cattle, including *S.* Dublin [120,121,122]. 

### 5.2. Prebiotics

Prebiotics are non-digestible carbohydrate substrates that selectively promote the growth of most of the beneficial or probiotic microflora when supplemented in the diet [123]. These fermentable carbohydrates mainly act in the lower intestine, resulting in the production of short-chain fatty acids and promote the growth of intestinal probiotic bacteria such as *Bifidobacterium* and *Lactobacillus* [124]. 

Studies conducted by Pourabedin et al. [125] revealed that the supplementation of mannan-oligosaccharides (MOSs) and xylo-oligosaccharides (XOSs) caused a significant reduction (1.6 and 1.0 log_10_ CFU/g, respectively) of the cecal colonization of *S.* Enteritidis. A study conducted by Fernandez et al. [126] revealed that 2.5% dietary supplementation of MOS resulted in reduced colonization of *Salmonella* spp. in the cecum of chicks. Supplementation of arabinoxylan oligosaccharides resulted in reduced colonization of *S.* Enteritidis in the cecum of broilers, and the supplementation also resulted in reduced shedding of the pathogen through the feces [127]. 

Similar to poultry, galacto-oligosaccharides and polysaccharides derived from seaweeds have been found to enhance the intestinal health of pigs when challenged with *S.* Typhimurium. The *S.* Typhimurium numbers were less in the cecum, colonic digesta, and fecal samples when the pigs were supplemented with seaweed-derived polysaccharides [128]. Another study conducted by Tanner et al. [129] also revealed that prebiotics such as fructo-oligosaccharides and galacto-oligosaccharides reduced *S.* Typhimurium in vitro when simulated with proximal colon conditions (38 °C, pH 6.0, retention time 9 h, and anaerobiosis) in the pig. 

Prebiotics act in different ways to bring about the desired effect in the host. Prebiotics modulate beneficial microorganisms in the gut, stimulate the host immune system, and reduce various virulence factors of the pathogen which are responsible for its colonization in the host. Prebiotics reduce pathogen attachment and invasion to the host intestinal epithelium [130]. Even though the prebiotics have a direct effect on the pathogens, most of the time prebiotics are used in combination with probiotics (synbiotics) to exclude pathogens in poultry and livestock. 

### 5.3. Plant-Derived Compounds

There is increasing interest in the use of natural compounds as antibiotic alternatives against foodborne pathogens [131,132,133,134,135,136,137,138]. Essential oils are volatile aromatic compounds obtained from different plant parts and are effective against foodborne pathogens, including *Salmonella*, in vivo and in vitro [133,134,135,139,140,141,142,143,144]. The essential oils have different components, and thereby use multiple mechanisms against pathogens. Therefore, the potential for developing resistance to essential oils and their ingredients is highly unlikely [145,146]. 

Plant-derived compounds such as *trans*-cinnamaldehyde and eugenol were found to be effective against *Salmonella* colonization in layer and broiler chickens [133,134]. Subinhibitory concentrations of *trans*-cinnamaldehyde and eugenol downregulated motility and invasion genes, and infeed supplementation of *trans*-cinnamaldehyde (0.5% and 0.75%) and eugenol (0.75% and 1.0%) resulted in more than 3 log_10_ CFU/g reductions in cecal colonization of *Salmonella* in broiler chicks. Also, *trans*-cinnamaldehyde (0.75%) and eugenol (1%) were highly effective against *S.* Enteritidis colonization in market-age broilers [134]. In a different study, it was reported that plant-derived molecules alone or in combination increased the sensitivity of multidrug-resistant *S.* Typhimurium DT104 towards antibiotics, ampicillin, chloramphenicol, streptomycin, sulfamethoxazole, and tetracycline [147]. The pathogen was susceptible to chloramphenicol, streptomycin, sulfamethoxazole, and tetracycline when used with thymol and *trans*-cinnamaldehyde. Similarly, carvacrol increased the susceptibility of the pathogen to sulfamethoxazole. However, a combination of *trans*-cinnamaldehyde, β-resorcylic acid, carvacrol, thymol, and eugenol resulted in a higher susceptibility of the pathogen to the tested antibiotics [147]. Another plant-derived molecule, namely carvacrol, at its subtherapeutic dose was found to be effective in reducing *S.* Typhimurium motility and its invasion of porcine epithelial cells [148]. 

Essential oils and their ingredients are generally recognized as safe (GRAS) flavoring agents and food additives. However, their use at higher concentrations in food may negatively impact the organoleptic qualities, including flavor and odor, thereby decreasing consumer acceptance. Therefore, a delicate balance exists between the selection of an effective concentration of these compounds against pathogenic microorganisms and their use in food systems [131]. Essential oils and their ingredients are widely used in meat products and fresh produce to reduce foodborne pathogens. Carvacrol, thymol, and eugenol are found to be effective against foodborne pathogens such as *Salmonella* spp. when used as post-chill dip treatment alone or in combination with high-carbon dioxide packaging on turkey breast cutlets [141,142]. Similarly, carvacrol, thymol, and eugenol were effective in reducing *Salmonella* attached to the tomato surface [149]. Cinnamon oil (0.8–1.0%) and olive extract (4–5%) were effective in reducing multidrug-resistant *S.* Typhimurium DT104 in ground pork [150]. These essential oils are also effective against *Salmonella* in beef products [131,151,152]. 

### 5.4. Organic Acids 

Medium- and short-chain fatty acids possess antibacterial efficacy against *Salmonella* spp. Short-chain fatty acids such as butyrate downregulate the invasion genes in *Salmonella* whereas propionate reduces *Salmonella* invasion [153]. A medium-chain fatty acid, caprylate (15 mM), was found to be effective against *S.* Typhimurium in a simulated cecal environment and caused more than 4 log_10_ CFU/g reduction of *Salmonella* [154]. Butyric acid was found to decrease intestinal colonization and fecal shedding of *S.* Typhimurium in pigs. Similarly, medium-chain fatty acids such as a caproic or caprylic acid (2 mM) downregulated virulence genes of *S.* Typhimurium such as *hilA* and *fimA* [155]. Short-chain fatty acids such as formic acid, acetic acid, propionic acid, and sorbic acid in combination with natural extracts were found to be effective in reducing cecal colonization and fecal shedding of *Salmonella* in market-age pigs [156]. 

A study conducted by Evans et al. [157] revealed that when medium-chain fatty acids were supplemented through feed, a 1 log_10_ reduction in the cecal colonization of *S.* Typhimurium was observed in turkey poults 3 days after inoculation. Also, supplementation of caprylic acid at 0.7% and 1.0% significantly reduced *Salmonella* after 5 days of challenge in chickens [158,159]. A study by Van Immerseel et al. [160] also revealed that caproic acid at 3 g/kg feed resulted in a significant reduction of *Salmonella* in the cecum of broilers. In these two studies, a reduction of *Salmonella* invasion to the liver and spleen were observed along with a reduction in the cecal colonization. Since short- and medium-chain fatty acids are effective against *Salmonella* and do not affect the performance of the livestock, these can be used as alternatives to antibiotics to control antibiotic-resistant *Salmonella* colonization in livestock species.

## 6. Ongoing Studies with Alternative Interventions against Multidrug-Resistant *Salmonella*

Multidrug-resistant *S.* Heidelberg is emerging as a significant pathogen causing foodborne disease outbreaks in the U.S. through contaminated poultry products. In 2011, the pathogen caused foodborne outbreaks through contaminated ground turkey products that resulted in 136 illnesses in 34 states [10]. The outbreak isolates were resistant to several commonly prescribed antibiotics, including ampicillin, streptomycin, gentamicin, and tetracycline [10,161]. In our lab, we are developing non-antibiotic interventions against this multidrug-resistant *Salmonella.* A dairy probiotic bacteria, namely *Propionibacterium freudenreichii*, was found effective against *Salmonella* spp., including the multidrug-resistant *S*. Heidelberg. A study conducted by Nair and Kollanoor-Johny [162] revealed that probiotic *P. freudenreichii* isolated from fermented dairy products was effective against major virulence factors of multidrug-resistant *S.* Heidelberg. Compared to the antibiotic-resistant parent strains, probiotic-treated *S.* Heidelberg showed reduced multiplication, motility, and adhesion on intestinal epithelial cells. Follow-up in vivo study revealed that supplementation of *P. freudenreichii* to 14-day turkey poults resulted in reduced pathogen colonization in the cecum [163]. Also, *P. freudenreichii* in combination with a mannanoligosaccharide prebiotic and a *Salmonella*-specific vaccine was found to be effective in reducing cecal colonization of *S.* Heidelberg in 7-week and 12-week old turkeys (unpublished data). In another study, we found that multiple combinations of probiotics (*Lactobacillus* of turkey-gut origin), prebiotic, and the *Salmonella* vaccine were effective in reducing *S.* Heidelberg colonization in the cecum of 14-day turkey poults. These treatments also reduced the *S.* Heidelberg invasion of the liver and spleen [164]. In addition, these combinations were also found to be effective in 7-week-old and 12-week-old-turkeys (unpublished data). 

We also found plant-derived compounds such as *trans*-cinnamaldehyde and pimenta essential oil were effective against multidrug-resistant *S.* Heidelberg isolated from ground turkey. Supplementation of *trans*-cinnamaldehyde through drinking water resulted in 4.5 log_10_ CFU/g reduction in cecal colonization and reduced invasion of *S.* Heidelberg to the liver and spleen of 14-day old turkey poults [165]. Pimenta essential oil was effective against *S.* Heidelberg attached to the turkey skin. Our studies revealed that pimenta essential oil was effective against the multidrug-resistant *S.* Heidelberg attached to the turkey skin and resulted in >2 log_10_ CFU/inch^2^ reduction at simulated chilling or scalding conditions during processing [166]. More studies are ongoing in the laboratory exploring the potential of antibiotic alternatives against multidrug-resistant enteropathogens. 

## 7. Conclusions and Future Directions

The issue of antibiotic resistance has resulted in far-reaching outcomes in human health and wellbeing due to the increased health care costs and productivity loss, and high proclivity towards acquiring other serious illnesses. A major issue with antibiotic resistance is that antibiotic-resistant clones of several major pathogens, including *Salmonella*, have been increasingly isolated from the food supply, including food animals, poultry, retail meat products, fresh produce, and seafood. All major resistance determinants, including those that confer resistance to β-lactams, extended spectrum β-lactams, fluoroquinolones, aminoglycosides, tetracyclines, and chloramphenicol, have been identified in various *Salmonella* serovars isolated from the food supply. It has become increasingly clear that antibiotic resistance will remain a significant hurdle to tackle in the near future. To address this issue, the FDA has issued the final rule to phase out antibiotics from production agriculture, curbing the use of clinically relevant antibiotics from production, and necessitating veterinary oversight on antibiotic use for therapy purposes in food animals and poultry. Responding to the situation, alternatives such as probiotics, prebiotics, phytobiotics, and others are being tested against drug-resistant pathogens, due to the broad spectrum of antimicrobial activity offered by these interventions. Ideally, the alternatives should not be toxic and should not result in residue build-up in the meat or eggs. It should be palatable to animals, stable in the gut, augment beneficial flora, and inactivate harmful pathogens. Additionally, these interventions will be tested for improved feed efficiency and growth without adversely affecting the environment [167]. Most importantly, they should not induce antimicrobial resistance in bacteria, including the beneficial gut microflora. Although studies targeting multiple serovars of *Salmonella* with these interventions are increasing, most of the studies are at their preliminary stages, warranting additional research to address significant gaps in the knowledge before recommending their use for improving preharvest and postharvest food safety. It will be a significant task to characterize, optimize, and scale-up these interventions to the level of potency and safety that antibiotics were providing in the past several decades. 
